# Conjunctival Vascular Metrics Using Automated Vessel Detection from Slit Lamp Images for Hyperemia Severity Assessment

**DOI:** 10.3390/diagnostics16132066

**Published:** 2026-07-01

**Authors:** Damon Wong, Yvonne Ng, Leila Sara Eppenberger, Eduard Toma, Radu Bucsan, Dan George Deleanu, Alina Popa Cherecheanu, Gerhard Garhöfer, Leopold Schmetterer

**Affiliations:** 1 Singapore Eye Research Institute, Singapore National Eye Centre, Singapore 168751, Singapore; damon.wong@duke-nus.edu.sg (D.W.); 2 SERI-NTU Advanced Ocular Engineering (STANCE) Program, Singapore 308232, Singapore; 3 Ophthalmology and Visual Sciences Academic Clinical Program (Eye ACP), Duke-NUS Medical School, Singapore 169857, Singapore; 4 Department of Ophthalmology, Carol Davila University of Medicine and Pharmacy, 050474 Bucharest, Romania; 5 Department of Ophthalmology, Emergency University Hospital, 050474 Bucharest, Romania; 6 Department of Clinical Pharmacology, Medical University of Vienna, 1090 Vienna, Austria; 7 Lee Kong Chian School of Medicine, Nanyang Technological University, Singapore 308232, Singapore; 8 School of Chemistry, Chemical Engineering and Biotechnology, Nanyang Technological University, Singapore 637459, Singapore; 9 Center for Medical Physics and Biomedical Engineering, Medical University of Vienna, 1090 Vienna, Austria; 10 Fondation Ophtalmologique Adolphe De Rothschild, 75019 Paris, France

**Keywords:** conjunctival hyperemia, hyperemia grading, vessel metrics, vessel segmentation, deep-learning

## Abstract

**Background/Objectives:** Conjunctival hyperemia is a common clinical finding in clinical practice; however there are significant differences between graders. Vessel detection using deep-learning approaches could enable more objective measures. We aimed to evaluate vascular metrics derived from automated vessel detection and compare these metrics with manual severity gradings. **Methods:** Slit lamp images from 139 glaucoma patients were included. Images from 103 participants were used as the primary development dataset and the remaining as a validation subset. The images were independently graded by two graders for conjunctival hyperemia using the Efron Grading Scheme. Conjunctival vessels were detected using an automated vessel detection pipeline based on semi-supervised learning. Vessel density, fractal dimension and tortuosity were calculated and compared with the manual Efron grades. **Results:** Grading of conjunctival hyperemia between the two graders were consistent (Spearman’s rho: 0.79; ICC: 0.79 [95%CI: 0.72–0.84]) but showed significant differences with a higher proportion of differences in the moderate grades. Of the vascular metrics, vessel density showed significant associations with the individual Efron grading and against the mean Efron grading (0.78, *p* < 0.001). Fractal dimension was significantly associated with the mean Efron grading (0.55, *p* < 0.001). Agreements were similar in the subset (vessel density, 0.80, *p* < 0.001; fractal dimension 0.62, *p* < 0.001). Vessel tortuosity showed lower agreements (<0.23). **Conclusions:** Vessel density and fractal dimension showed significant associations with manual Efron gradings. These metrics could be potentially used to enable more objective and interpretable measures of conjunctival hyperemia severity.

## 1. Introduction

Conjunctival hyperemia, also commonly referred to as bulbar hyperemia, is one of the most common clinical findings in ophthalmic practice [1,2]. It presents as a visible redness in the conjunctiva due to the inflammation of the conjunctival vessels [3]. The condition is often associated with early signs of dry eye syndrome [4], reactions to environmental allergens, or adverse reactions from the instillation of eye drops in treatment interventions [5,6,7]. With the increasing prevalence of hyperemia [8], assessment of its severity has become a growing need to better monitor treatment efficacy and adverse reactions in the development of novel interventions.

Currently, several image-based grading schemes have been introduced for the assessment of conjunctival hyperemia [9]. One of the most widely used and accepted in research and clinical practice is the Efron Scale [10], comprising of several reference illustrations. The Efron scale was originally developed to evaluate ocular complications arising from contact lens wear at five severity levels and has since been adopted for other ocular conditions. Other well-known scales include the grading scheme developed by McMonnies and Chapman-Davies (MC-D) [11], and the Validated Bulbar Redness Scale (VBR) [12]. However, significant inter-observer challenges can occur with these manual grading systems, which are based on subjective human assessment in comparison with reference images. Grades can vary based on expertise and training, and the limited discretization provided by traditional scales can hinder the documentation of minor changes [13,14,15].

Inter and intra-observer variability in grading based on manual subjective assessments has motivated the need for more objective and interpretable grading for the severity of hyperemia based on vascular appearance. While direct prediction of severity labels using deep learning has been explored, this approach is limited by poor transparency and interpretability, undermining its clinical applicability [16]. Recently, deep-learning-based frameworks for the detection of vascular structures have demonstrated a high level of fidelity in various medical fields, including cardiology [17], radiology [18] and ophthalmology [19], often showing reduced variability and improved detection of microvasculature compared to manual labels. Vascular structures detected from deep-learning frameworks could enable the development of metrics for more objective assessments of conjunctival hyperemia, which can benefit the clinical management. There remains however limited data on such metrics.

The objective of this study was to compare Efron gradings from two independent graders to determine inter-observer variability and evaluate several vascular metrics derived from conjunctival vessel segmentations obtained using a deep-learning-based vascular segmentation model against manual hyperemia grading.

## 2. Materials and Methods

### 2.1. Participants

This work is based on the retrospective analysis of data collected in an earlier study of glaucoma patients undergoing eyedrop treatment conducted at the Medical University of Vienna, Austria. The study was conducted in accordance with the Declaration of Helsinki and approved by the Ethics Committee of the Medical University of Vienna (approval code: EC No 2003/2024; approval date: 9 December 2024). Informed consent was obtained from all subjects involved in the study.

### 2.2. Image Acquisition and Conjunctival Hyperemia Grading

Slit lamp images of the conjunctiva were acquired from the study participants and graded for severity of hyperemia using the Efron Grading Scale. The Efron Grading Scale ranges from grade 0 (Normal) to grade 4 (Severe), with a reference illustration provided at each severity scale. Ungradable images due to poor image quality were excluded. Two graders were masked to participant characteristics and were asked to independently evaluate the severity of hyperemia based only on the presented images using the Efron Grading Scale.

### 2.3. Conjunctival Vessel Segmentation Pipeline

**Figure 1:**
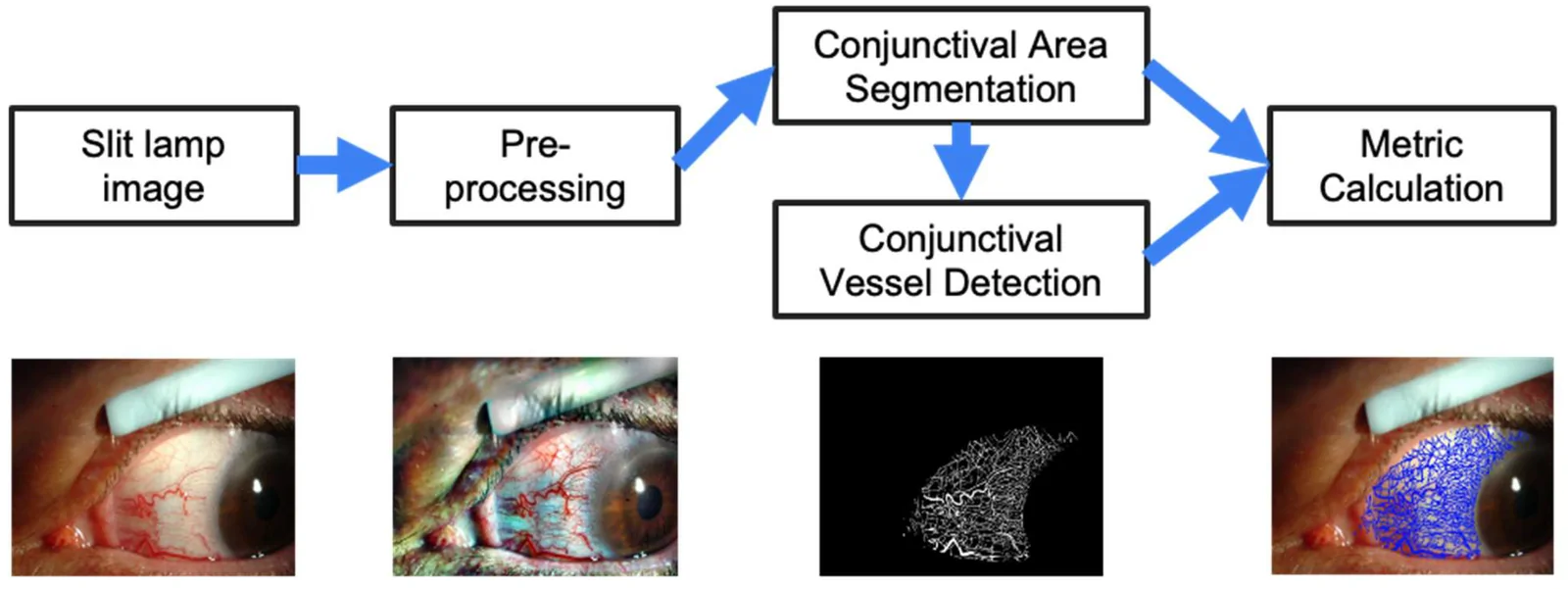
Conjunctival vessel detection pipeline. An input slit lamp image first undergoes preprocessing to enhance contrast. Conjunctival area and enclosed vessels are detected using the semi-supervised segmentation models. Vessel metrics are calculated based on the detected vascular structures within the conjunctiva.

We applied a segmentation pipeline for the detection of conjunctival vessels (Figure 1). The pipeline is based on a semi-supervised-learning cross-teaching approach which includes unlabeled data in addition to manually annotated ground truth labels [20]. Details of the model have been previously reported [21]. Briefly, the architecture leverages cross-training between different learning paradigms to improve segmentation performance by sharing pseudo-labels, reducing errors. In the cross-teaching framework, a convolutional neural network (U-Net) and attention-based network (Swin U-Net), selected based on architecture diversity, were jointly trained to distillate local spatial and semantic features between the networks during training. A schematic of the framework is provided in Supplementary Figure S1. To minimize computational complexity for use in practice, we used the cross-trained U-Net which has reduced computational requirements (11 GFlops) compared to Swin U-Net (24 GFlops). We used this approach to develop two segmentation models, one for conjunctiva segmentation and the other for segmenting vascular structures within the region of interest. Contrast Limited Adaptive Histogram Equalization (CLAHE) was applied to all images to enhance overall contrast. For the conjunctiva dataset, data augmentation techniques including horizontal flipping, vertical flipping and brightness variation were applied. Once the conjunctiva mask was obtained, regions where vessels were present were extracted. For vessel segmentation, a sliding window approach was used to generate patches of 256 × 256 pixels with a 50% overlap. No image resizing of the tiles was performed to preserve fine vascular structure details. Patch stitching was then applied to reconstruct the overall vessel structure generating the predicted binary vessel mask. Specific training details have been reported previously [21]. The models were developed on a workstation equipped with a RTX 3090 GPU.

### 2.4. Metrics

Different metrics were employed to further study the structural complexity and changes in the vessel curvature of the conjunctiva blood vessels from the extraction of segmented vessels. These were selected based on their utility in the characterization of retinal vasculature [22]. In this study, quantitative measurements relating to percentage of occupation by vessels (vessel density), complexity of vessel structure (fractal dimension) and vessel trajectory (vessel tortuosity) were calculated to quantify their correlation with Efron severity grading.

### 2.5. Vessel Density

Vessel density (*V_D_*) is a measure of the percentage of vessel pixels within the conjunctival region of interest. It reflects the overall vascular coverage within the conjunctival region, capturing how densely the blood vessels are distributed across the tissue. A higher *V_D_* indicates that blood vessels cover a large proportion of the region, reflecting increased vascular coverage across the conjunctiva surface. Increased inflammation causes more vessels to become visible resulting in higher *V_D_* which could be associated with higher Efron severity grades. *V_D_* was directly derived from the predicted binary vessel mask generated by the segmentation model. Only pixels within the conjunctiva boundary were considered in the computation. *V_D_* was quantified as the ratio of the total number of vessel pixels (*N_V_*) to the total number of pixels within conjunctiva mask (*N_T_*), as expressed in the equation below.

(1) $$V_{D}=\frac{N_{V}}{N_{T}}$$

### 2.6. Fractal Dimension

Fractal dimension (*F_D_*) is a quantitative measure of the overall structural complexity and self-similarity of the vascular network. It reflects how densely vessels occupy space across different spatial scales. Different fractal dimension variations have been reported in literature [22]. For our study, we used the definition of capacity fractal dimension which measures the completeness of the vessel network occupying the space across multiple scales and has also been used in prior studies [23]. Higher *F_D_* values indicate denser and more complex vascular network which is expected to correspond with increasing conjunctival hyperemia severity along the Efron grading scale. *F_D_* was evaluated based on box-counting to measure the overall vascular network by quantifying the completeness of vessels occupying the space within each bounding box. Detected vessels were overlaid with square grids of varying box size ϵ with different scales. For each scale, the number of boxes *N*(*ϵ*) containing the vessels was calculated. This was repeated across multiple scales. A log-log plot of *N*(*ϵ*) versus 1/*ϵ* was computed as shown in the formula below:

(2) $$F_{D}=\underset{\epsilon \to 0}{\lim}\frac{\ln N(\epsilon )}{\ln\left(1/\epsilon \right)}$$

This metric reflects the global vessel density and space-filling capacity of the vascular structures in each image, with a greater the vessel coverage and branching complexity corresponding to higher *F_D_* values.

### 2.7. Vessel Tortuosity

Vessel tortuosity (*V_T_*) is a metric that quantifies the degree of curvature and winding of individual blood vessel segments, defined as the ratio of actual pathlength of vessel segment to the Euclidean distance between its endpoints. Increased curvature and twisting of the vessel trajectory is represented as an increase in tortuosity. This metric allows the study of the changes in vascular structures across all vessel segments, providing a single measurement for correlation with Efron severity grades. To calculate the tortuosity, skeletonization was applied to the detected vessel, and a graph-based approach was applied to generate the nodes and vessel segments, identifying the start and end points of each individual vessel segment. Tortuosity was calculated based on the sum of pathlength (*L_C_*) divided by the Euclidean distance from the start and end point for each vessel (*L_x_*).

(3) $$V_{T}=\frac{L_{c}}{L_{x}}$$

Mean vessel tortuosity in each image was calculated by averaging the tortuosity values across all vessel segments.

### 2.8. Statistical Analysis

Continuous variables are presented as mean and standard deviation (SD), with the Efron grades presented as median and inter-quartile range (IQR). Inter-grader agreement for the manual Efron grades was evaluated using Spearman’s rank correlation coefficient *ρ*, weighted Kappa (*κ_w_*), which is a variation of Cohen’s kappa for ordinal labels, and intra-class correlation coefficients (ICC) with 95% confidence intervals. Limits of agreement were evaluated using Bland–Altman analysis, and Wilcoxon signed-rank tests were used to evaluate differences in the Efron grades. Strength of associations between the vascular metrics against the individual Efron grades and the average Efron grades were evaluated using Spearman’s rank correlation coefficient. Statistical analyses were performed using the Scikit-learn Python library ver 1.6. *p* values less than 0.05 were deemed statistically significant.

## 3. Results

### 3.1. Efron Grading

**Figure 2:**
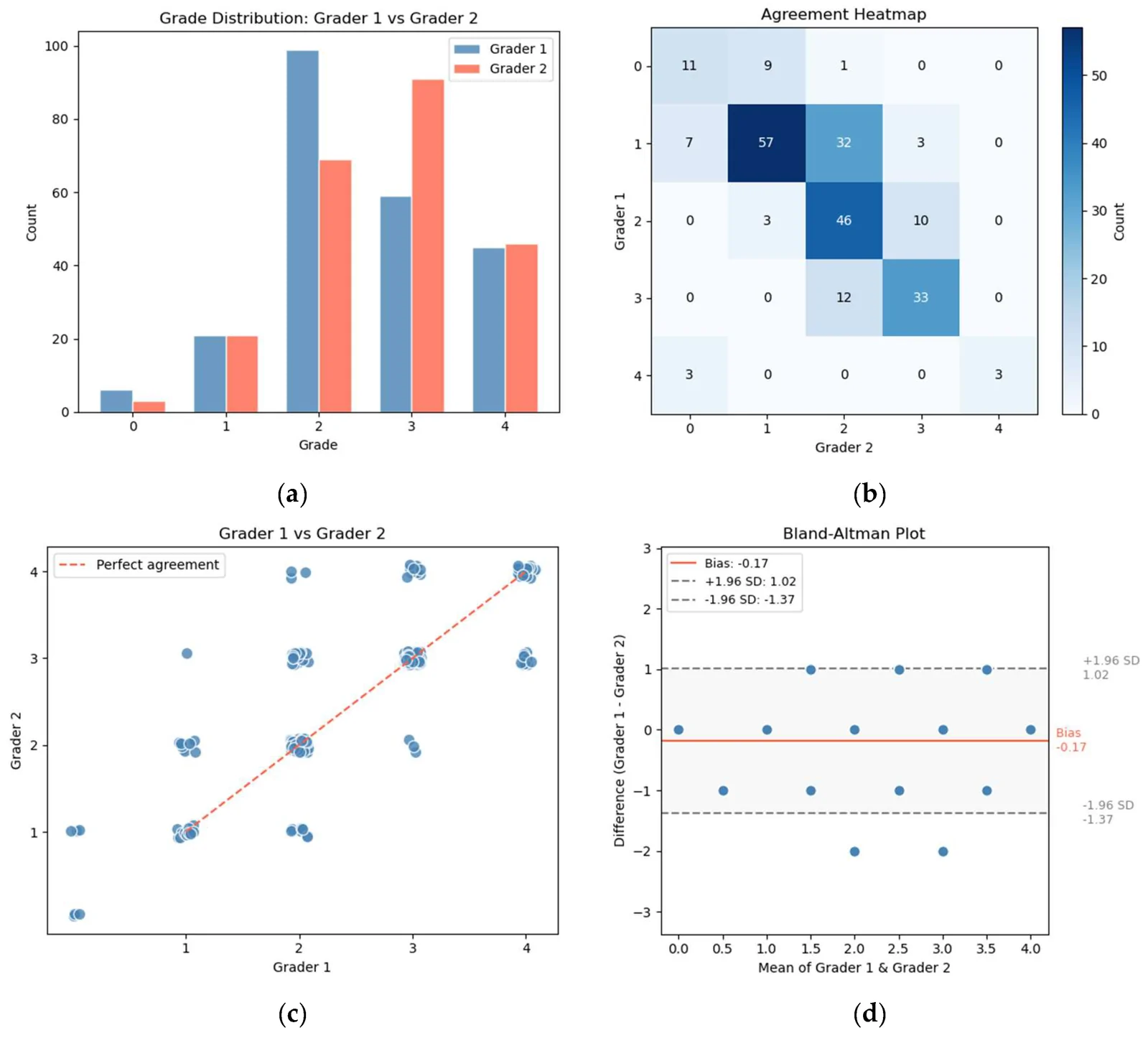
Comparisons between the manual Efron grades in the development dataset. Subplots show (**a**) histograms of the distributions, (**b**) inter-observer agreement heatmap, (**c**) scatter plot between the graders and (**d**) Bland–Altman plot showing limits of agreement.

The development dataset included 230 slit lamp images from 103 participants with glaucoma (51% males), with an average age of 66.7 ± 10.6 years, and a subset of data comprising of 164 images from 36 glaucoma patients (63.9 ± 14.0 years, 36% males) were used as an independent validation subset. Distributions of the Efron grading outcomes from each grader from the development dataset is provided in Figure 2a. The first grader had a median Efron grade of 2.0 (IQR 1.0–3.0), with the second grader having a median Efron grade of 3.0 (IQR 2.0–4.0). In the images from the subset, the first and second graders had manual Efron distributions of 2.0 (IQR 2.0–3.2) and 2.0 (IQR 1.0–3.0), respectively.

### 3.2. Inter-Grader Comparison

In the development dataset, the two graders were generally consistent in rank (Spearman’s *ρ*: 0.79; weighted Kappa *κ_w_*: 0.66; ICC: 0.79 [95%CI: 0.72–0.84]). However, differences between the Efron scores from the two graders were significantly different (Wilcoxon signed-rank test, *p* < 0.001), with a mean difference of 0.17 (95% Limits of Agreement [LoA]: 1.19). Differences between the graders largely occurred in the more moderate Efron severity grades between 2 and 3 (Figure 2b–d). In the validation subset, the graders showed larger differences in their ratings, with a mean difference of 0.71 (95% LoA: 1.33; weighted Kappa *κ_w_*: 0.34; ICC: 0.59 [95%CI: 0.04–0.81]), although the ranks remained consistent between the two graders (Spearman’s *ρ*: 0.77).

### 3.3. Conjunctival Vessel Detection and Metrics

**Figure 3:**
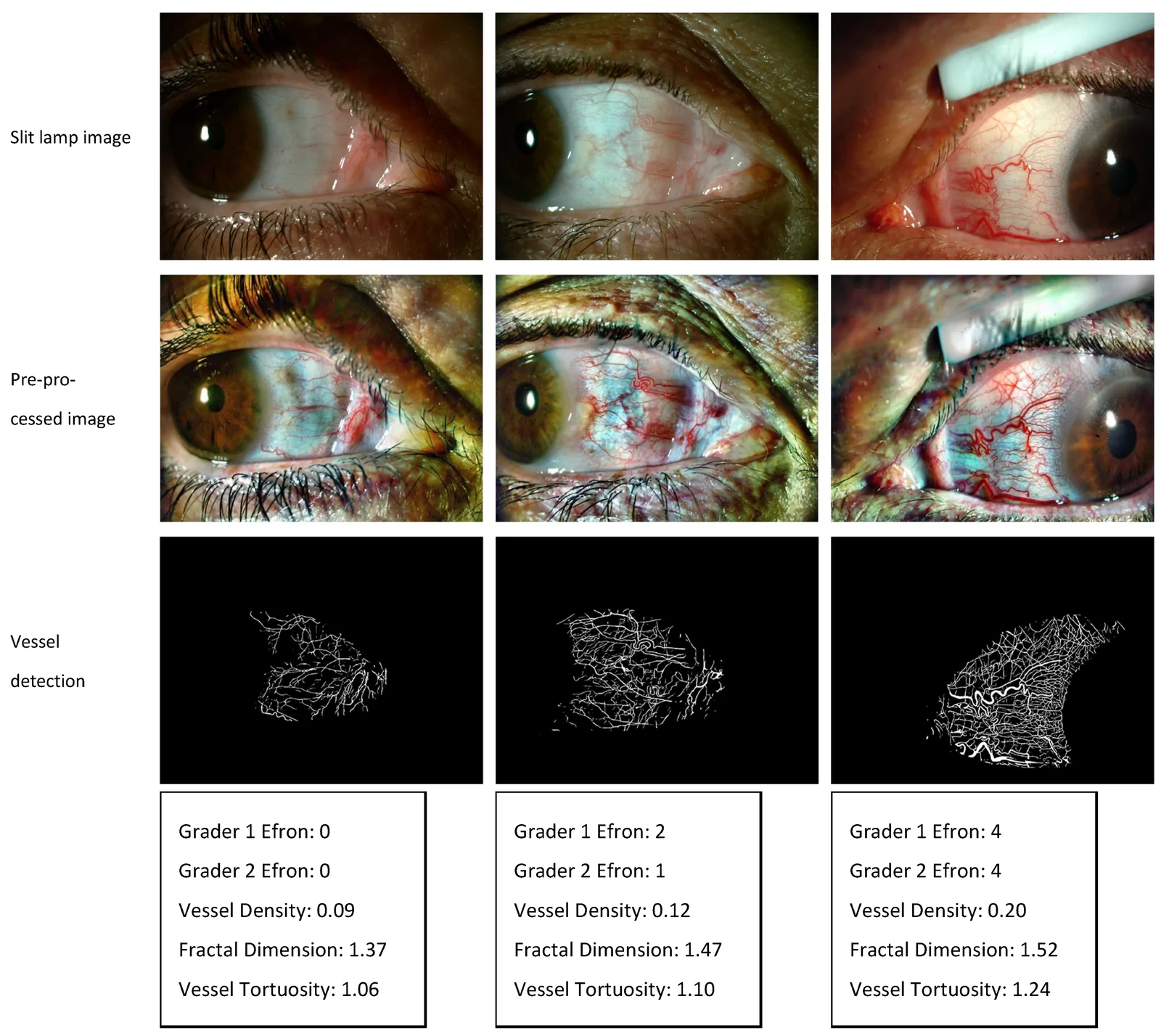
Results of vessel detection and metrics using the segmentation pipeline. First row: slit lamp images; second row: pre-processed images; third row: detected vessels. Middle column shows an example of an image with differing Efron grading between the two graders.

We applied our conjunctival vessel segmentation framework on the images in this study directly without further fine tuning or adjustments. Each image was processed in approximately 40 s. Using the detected conjunctival vessels, we calculated several vascular metrics representing the vessel density (*V_D_*), fractal dimension (*F_D_*) and tortuosity (*V_T_*). Examples of the different metrics and Efron grades are shown in Figure 3. In the development dataset, the mean ± SD vessel density was 0.13 ± 0.03 (SD), mean ± SD fractal dimension was 1.49 ± 0.06, and mean ± SD vessel tortuosity was 1.39 ± 0.40 over the dataset. For the subset, the mean ± SD *V_D_* was 0.16 ± 0.03, mean ± SD *F_D_* was 1.58 ± 0.03, and mean ± SD *V_T_* was 1.54 ± 0.28.

### 3.4. Comparison of Vessel Metrics with Efron Grading

We compared the association of these vascular metrics with the individual Efron gradings and the mean Efron grading between the graders in the development dataset (Table 1) and subset (Table 2). Vessel densities showed better associations with the manual Efron grades compared to the other metrics, achieving a Spearman’s *ρ* of 0.78 in the development dataset with the mean Efron grading, comparable to the associations between the Efron grades from the two graders (Spearman’s *ρ*: 0.79). Similar results were observed in the validation subset for vessel density against the mean grading (Spearman’s *ρ*: 0.80) compared the inter-grader agreement (Spearman’s *ρ*: 0.77).

**Figure 4:**
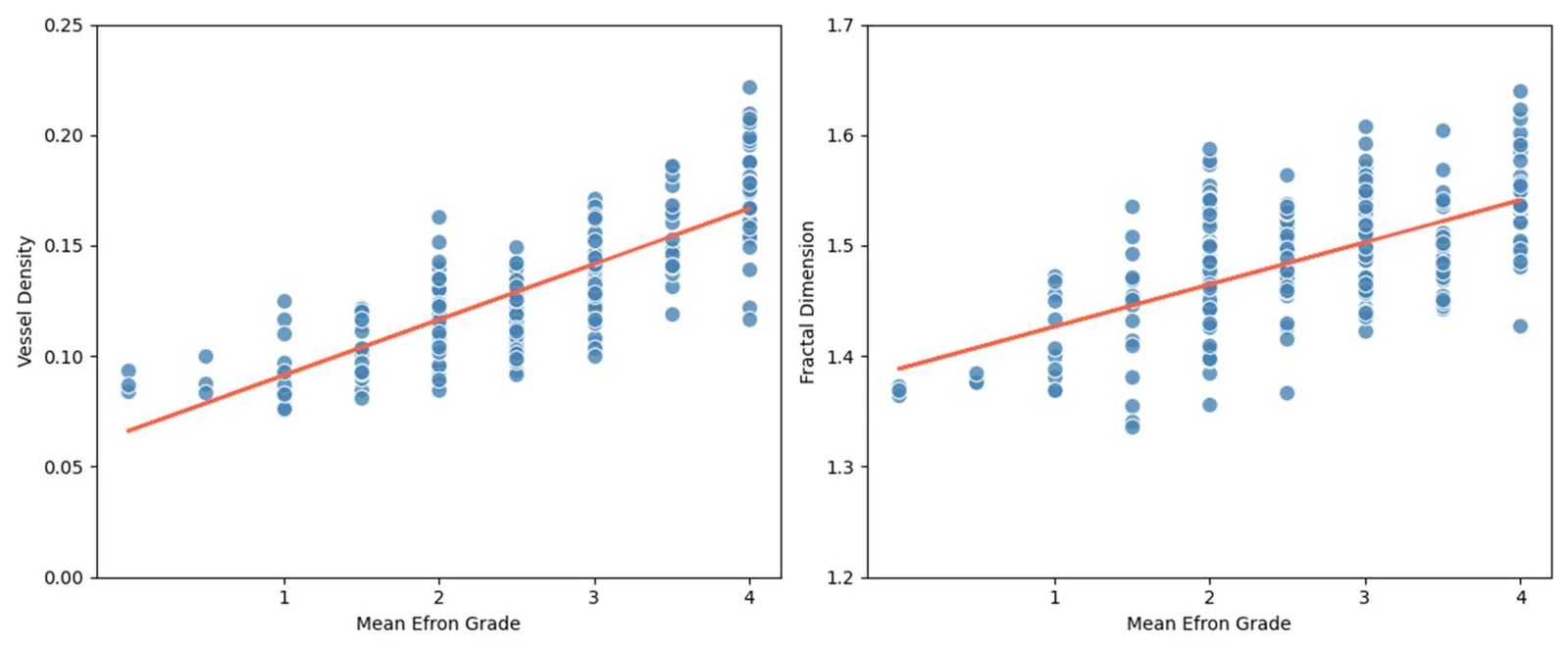
Scatter plots of vessel density (**left**) and fractal dimension (**right**) against the mean Efron grades between the two graders for the development dataset. Red line indicates the linear gradient between the vascular metrics and the mean Efron grades.

**Figure 5:**
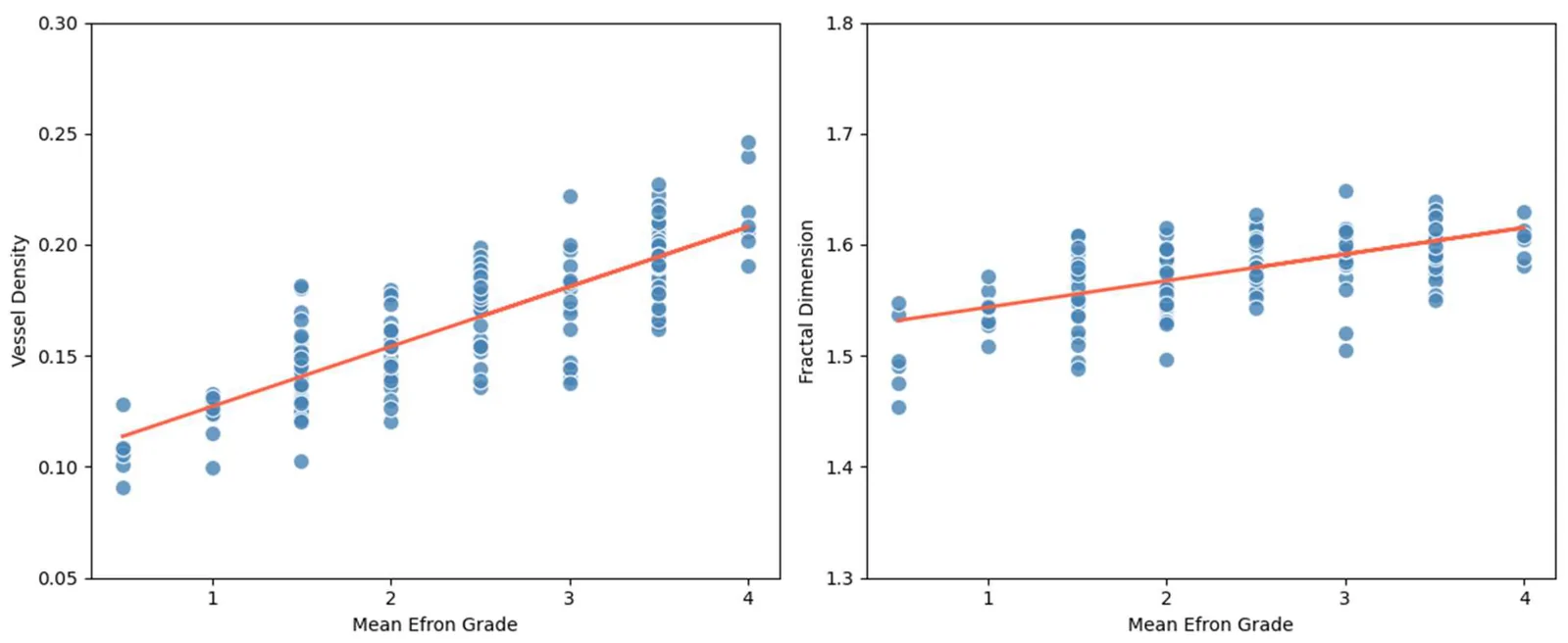
Scatter plots of vessel density (**left**) and fractal dimension (**right**) against the mean Efron grades between the two graders for the validation subset. Red line indicates the linear gradient between the vascular metrics and the mean Efron grades.

Scatter plots of the vessel density and fractal dimension against the mean Efron grades are presented in Figure 4 for the development dataset and Figure 5 for the validation subset. Fractal dimensions showed lower associations but were still significant. In contrast, vessel tortuosity showed only weak associations with the manual grades (*ρ* < 0.25) and was not consistently significant across the development and validation test sets.

## 4. Discussion

This study evaluated the agreement between automatically generated vascular metrics and manual Efron grading in a cohort of glaucoma patients. Variability between manual Efron grading performed by two independent human graders was assessed and compared to metrics derived from automated detected conjunctival vessels obtained using a vessel segmentation approach based on semi-supervised learning. Efron gradings between the two graders were significantly associated (Spearman’s *ρ* 0.79, *p* < 0.001), showing that assessments of severity were largely ranked similarly. However, between the graders, the analysis found significant differences and limits of agreement of 1.19 grades, suggesting that Efron grades from different raters were not interchangeable. Of the vascular metrics, the vessel density and fractal dimension showed significant associations with the Efron gradings. The relationship between the vessel density and mean Efron grades between the two graders achieved an association of 0.78, which was comparable to the inter-observer comparisons. Similar results were observed in the validation subset. These results suggest that vessel metrics could potentially be used for evaluating the severity of conjunctival hyperemia. The main advantage of our study is the use of automatically generated vascular metrics from a deep-learning vascular detection approach and the availability of manual grading from two independent graders.

The assessment of conjunctival hyperemia is often complicated by the choice of severity grading schemes and inherent inter-observer variability. Although these grading schemes are largely consistent when ranking severity levels, there is limited compatibility [9,14]. Grades obtained with one grading scheme are not directly interchangeable with those from another [24], which can complicate the comparison of clinical outcomes from studies which have adopted differing grading schemes. This challenge is further compounded by inter-observer variance even when the same grading scheme is adopted, which can be due to differences in training and experience [24]. In our study, inter-observer annotations were largely consistent and correlated; however differences of up to two grades were observed, which could have clinical implications for intervention management and clinical trial outcome. Objective metrics representing interpretable characteristics, which are less vulnerable to observer bias and constrained to specific schemes are appealing to address these current limitations [21,25]. In our study, vascular measures derived from deep-learning-based segmentations showed strong associations with the Efron gradings and were comparable to inter-observer agreement. These metrics, which were calculated from automated conjunctival vessel detection in input slit images, may represent a more objective measure of severity and vascular characteristics that can be used for clinical assessment in the management of conjunctival hyperemia and also act as biomarkers for clinical trial outcomes. This motivates further studies to evaluate the development of further novel metrics to better assess hyperemia severity.

One of the main clinical characteristics of increased conjunctival hyperemia severity is increased apparent redness in the conjunctiva, due to dilation of blood vessels on the ocular surface from ocular inflammation. This visible symptom is often presented as findings of ‘red eye’ in the clinics and correlates to increased visibility of vascular structures on the ocular surface. In our study, among the three metrics, we found that the vessel density consistently showed better associations with the Efron gradings compared to the other metrics. This aligns well with the physiological interpretation of conjunctival redness, since it can be intuitively understood that increased redness stems from a higher visibility of vascular structures and a corresponding increase in the density of vascular structures over the conjunctiva. Our study also found that vessel fractal dimension showed a moderate level of association with the hyperemia gradings. Mathematically, fractal dimension is a derived geometric pattern that is a measure of vessel complexity. In ophthalmology, the use of fractal analysis has been proposed for the characterization of the branching patterns for retinal vasculature from retinal photographs [23], and its associations with cardiovascular risk [26], diabetes [27] and other systemic conditions [28] have been well-studied. However, it has yet to be fully explored in conjunctival hyperemia. In our study, the weaker association of hyperemia severity to fractal dimension compared to vessel density may be surmised to be due to increased irregularity of vascular structures coupled with increased visibility of the microvasculature in more severe hyperemia, which can confound associations in hyperemia. In contrast, the analysis of fractal dimension in retinal vasculature benefits from a more regular branch pattern and better defined major vascular arcades. Nevertheless, vessel density and fractal dimension provide two views of vascular structures in hyperemia that may provide additional complementary information.

Vessel tortuosity was not found to have significant associations with hyperemia, although it has been shown to have strong associations with cardiovascular risk [29] and diabetic retinopathy [30,31], similar to fractal dimension. In retinal vessel applications, the architecture of the retinal vasculature is relatively well-defined, with a clear origin at the optic nerve and increasing branching orders propagating from the major arcades. Typical retinal tortuosity analyses such as in the Singapore “I” Vessel Analysis (SIVA) [32] also define an analysis zone approximately 0.5–2.0 disc diameters from the optic disc for standardized comparisons. In conjunctival vessel analysis, particularly in increasing hyperemia, the determination of a clear vascular tree architecture is more complex, and unlike retinal vessel analysis, it can be challenging to define a standardized segment of the conjunctival architectures for analysis. Fractal dimension analysis is applied to the whole image and is less dependent on the definition of comparable vessel segments, which may explain its better association with hyperemia severity compared to vessel tortuosity.

There are several limitations of our study. Manual grading was only performed using the Efron Grading Scheme, and comparisons of the vascular measures against other grading schemes were not evaluated. The cohort was composed of only Caucasian participants with glaucoma, and the applicability of our findings to other ethnicities or other ocular conditions remains to be evaluated. Further, only images of acceptable quality were included for grading and analysis in our study; images acquired with poor quality were excluded. The robustness of our results in images acquired under non-optimal clinical conditions and different illumination was not explored. Our study was limited to vascular metrics which have been evaluated in the retina but heretofore not widely explored in conjunctival hyperemia analysis; further studies may include novel parameters more specific to the condition. Current results are obtained from a model developed on a research environment with a dedicated GPU. Processing requirements may differ for implementation on practical hardware systems, which can be estimated from prior studies [33].

Vascular metrics derived from automated deep-learning vessel detection showed significant associations with Efron grading performed manually in our study. This could lead to the development of more objective measures for improving the assessment of conjunctival hyperemia and management of the condition. Further studies are required to extend the applicability of our findings.

## Figures and Tables

**Table 1:** Comparison of vascular metrics against Efron grading in the development dataset.

Efron Grading	Vessel Density (*V_D_*)	Fractal Dimension (*F_D_*)	Vessel Tortuosity (*V_T_*)
Grader 1	**0.81, *p* < 0.001**	**0.52, *p* < 0.001**	0.11, *p* = 0.087
Grader 2	**0.67, *p* < 0.001**	**0.53, *p* < 0.001**	**0.15, *p* = 0.026**
Mean Efron Grade ^#^	**0.78, *p* < 0.001**	**0.55, *p* < 0.001**	0.13, *p* = 0.052

Values are presented as Spearman correlation coefficients of the metrics against the grades. Significant associations (*p* < 0.05) are shown in bold. ^#^ Mean Efron grades were calculated as the arithmetic mean of the grades assigned to each image by the graders.

**Table 2:** Comparison of vascular metrics against Efron grading in the validation subset.

Efron Grading	Vessel Density (*V_D_*)	Fractal Dimension (*F_D_*)	Vessel Tortuosity (*V_T_*)
Grader 1	**0.82, *p* < 0.001**	**0.66, *p* < 0.001**	**0.18, *p* = 0.022**
Grader 2	**0.68, *p* < 0.001**	**0.50, *p* < 0.001**	**0.23, *p* = 0.003**
Mean Efron Grade ^#^	**0.80, *p* < 0.001**	**0.62, *p* < 0.001**	**0.22, *p* = 0.004**

Values are presented as Spearman correlation coefficients of the metrics against the grades. Significant associations (*p* < 0.05) are shown in bold. ^#^ Mean Efron grades were calculated as the arithmetic mean of the grades assigned to each image by the graders.

## Data Availability

The data presented in this study are available on request from the corresponding due to patient confidentiality, privacy considerations, and institutional data protection policies.

## Supplementary Materials

The following supporting information can be downloaded at: https://www.mdpi.com/article/10.3390/diagnostics16132066/s1, Supplementary Figure S1. Schematic overview of the semi-supervised segmentation framework.
