# Correction: Wong et al. Conjunctival Vascular Metrics Using Automated Vessel Detection from Slit Lamp Images for Hyperemia Severity Assessment. *Diagnostics* 2026, *16*, 2066

**DOI:** 10.3390/diagnostics16162614

**Published:** 2026-08-18

**Authors:** Damon Wong, Yvonne Ng, Leila Sara Eppenberger, Eduard Toma, Radu Bucsan, Dan George Deleanu, Alina Popa Cherecheanu, Gerhard Garhöfer, Leopold Schmetterer

**Affiliations:** 1 Singapore Eye Research Institute, Singapore National Eye Centre, Singapore 168751, Singapore; damon.wong@duke-nus.edu.sg (D.W.);; 2 SERI-NTU Advanced Ocular Engineering (STANCE) Program, Singapore 308232, Singapore; 3 Ophthalmology and Visual Sciences Academic Clinical Program (Eye ACP), Duke-NUS Medical School, Singapore 169857, Singapore; 4 Department of Ophthalmology, Carol Davila University of Medicine and Pharmacy, 050474 Bucharest, Romania; 5 Department of Ophthalmology, Emergency University Hospital, 050474 Bucharest, Romania; 6 Department of Clinical Pharmacology, Medical University of Vienna, 1090 Vienna, Austria; 7 Lee Kong Chian School of Medicine, Nanyang Technological University, Singapore 308232, Singapore; 8 School of Chemistry, Chemical Engineering and Biotechnology, Nanyang Technological University, Singapore 637459, Singapore; 9 Center for Medical Physics and Biomedical Engineering, Medical University of Vienna, 1090 Vienna, Austria; 10 Fondation Ophtalmologique Adolphe De Rothschild, 75019 Paris, France

In the original publication [1], the funder Laboratoires Théa was not included. The corrected Funding and Conflicts of Interest appear below.

**Funding:** The financial support was provided by Laboratoires Théa.
**Conflicts of Interest:** The authors declare that this study received funding from Laboratoires Théa. The funder was not involved in the study design, collection, analysis, interpretation of data, the writing of this article or the decision to submit it for publication.

The authors state that the scientific conclusions are unaffected. This correction was approved by the Academic Editor. The original publication has also been updated.
