# Supplementary figures and images for: Conjunctival Vascular Metrics Using Automated Vessel Detection from Slit Lamp Images for Hyperemia Severity Assessment
Source: Diagnostics (Basel). 2026 Jul 1;16(13):2066. doi: 10.3390/diagnostics16132066 (PMC13359948; doi:10.3390/diagnostics16132066)

Supplementary Figure S1. Schematic overview of the semi-supervised segmentation framework

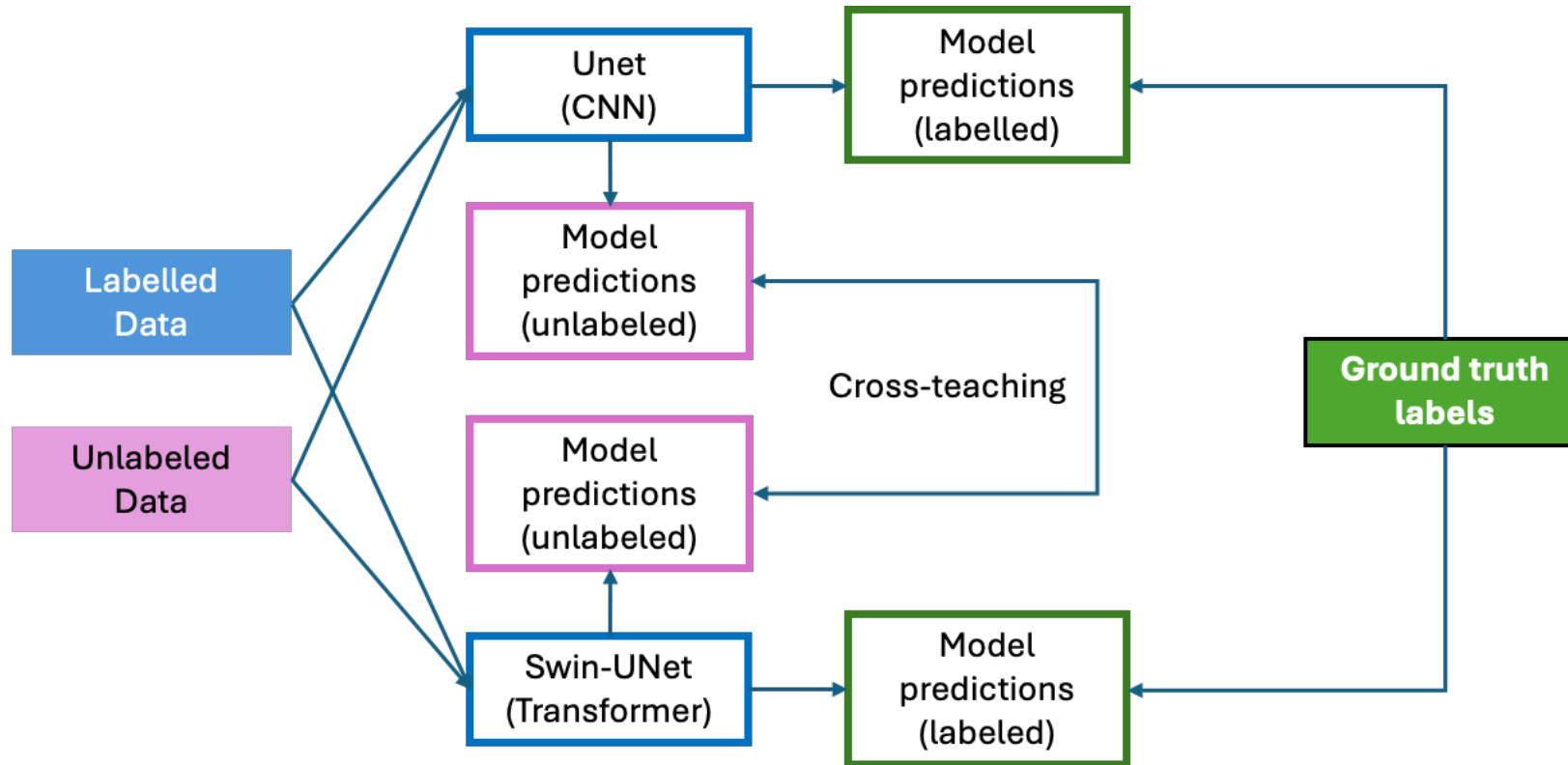

Supplement: Supplementary file 1 [file diagnostics-16-02066-s001.zip › diagnostics-4324473-supplmentary.pdf]
